# Giant electrocaloric and energy storage performance of [(K_0.5_Na_0.5_)NbO_3_]_(1−*x*)_-[LiSbO_3_]_*x*_ nanocrystalline ceramics

**DOI:** 10.1038/s41598-018-21305-0

**Published:** 2018-02-16

**Authors:** Raju Kumar, Satyendra Singh

**Affiliations:** Special Centre for Nanoscience, Jawaharlal Nehru University, New Delhi, 110067 India

## Abstract

Electrocaloric (EC) refrigeration, an EC effect based technology has been accepted as an auspicious way in the development of next generation refrigeration due to high efficiency and compact size. Here, we report the results of our experimental investigations on electrocaloric response and electrical energy storage properties in lead-free nanocrystalline (1 − x)K_0.5_Na_0.5_NbO_3_-xLiSbO_3_ (KNN-xLS) ceramics in the range of 0.015 ≤ x ≤ 0.06 by the indirect EC measurements. Doping of LiSbO_3_ has lowered both the transitions (T_*C*_ and T_*O*–*T*_) of KNN to the room temperature side effectively. A maximal value of EC temperature change, Δ*T* = 3.33 K was obtained for the composition with x = 0.03 at 345 K under an external electric field of 40 kV/cm. The higher value of EC responsivity, *ζ* = 8.32 × 10^−7^ K.m/V is found with COP of 8.14 and recoverable energy storage of 0.128 J/cm^3^ with 46% efficiency for the composition of x = 0.03. Our investigations show that this material is a very promising candidate for electrocaloric refrigeration and energy storage near room temperature.

## Introduction

An enormous amount of global energy uses for refrigeration, processing plants and air-conditioners are mainly based on vapor compression technology based on a century ago developed mechanical compressor. Due to bulk sizes, complexity, non-capability of scaling down for modern technologies such as for electronic chips, mobile devices, etc, the traditional technology is not very effective and also the coolant are non-environmental friendly^1–3^. In terms of the cooling capacity the coefficient of performance (COP), stated as the heat extracted from the system in each unit of the electrical energy consumed is low for existing technology.

The alternative technology for eliminating the compressor, vapor refrigerant coolant and with the high COP, is the solid state based refrigeration technology. An active research in material science is for the search of caloric materials for applying in proposed prototype solid-state cooling devices which works on the electrocaloric effect^4–6^. The electrocaloric effect (ECE) is the adiabatic change entropy/temperature induced by the change in polarization due to a sudden operation of the electric field in ferroelectric materials^7–9^. Ferroelectric materials exhibit spontaneous polarization and when it comes under the operation of an external electric field, the electric dipoles change the orientation from disorder to order state. The entropy correlated with the polarization decreases. Since a conclusive entropy of the system is constant, to compensate the changes in the material system the lattice entropy increases which results in the increment of temperature (ECE). Polar nano-domains are randomly oriented in the ferroelectrics, multiple possible orientation leads to the enhanced ECE^10^ and also it is higher at the phase transition due to opposite dipoles in a unit cell. In terms of energy barrier, at critical point the energy barrier reduced which fall in between different phases, since more than one polar phase exists at this point and the entropy increased which enhance ECE.

Among all nanostructures, thin film can produce high ECE, but the electrocaloric strength (|Δ*T*|/|Δ*E*|) which is the main factor to decide the strength over applied field reduces, in bulk ceramics it is higher and more promiseable structure^11–14^. The colossal electric fields are the reason for high ECE in thin films that can be applied till the breakdown field, the cooling effect and specific density are low. The cooling strength of bulk ceramics are of high value but the observed ECE is sufficiently low for practical purposes in commercial refrigeration technologies and the reason behind is the low dielectric strength. So, the search for achieving high polarization ceramics with high dielectric strengths in the broad temperature range around room temperature is one of the most emerging fields of condensed matter and material science. The EC refrigeration based on ECE has shown significant potential for developing the next generation cooling technology due to high efficiency, easy miniaturisation and low cost for the replacement of traditional vapor compression technology^15–17^.

However, the giant ECE have been observed for lead-based material which is again hazardous and carcinogenic. In order to overcome this problem lead-free material like (Na_0.5_Ba_0.5_)TiO_3_^18,19^, BaTiO_3_^20–23^, (K_0.5_Na_0.5_)NbO_3_^9,13,24^, Ba_0.65_Sr_0.35_TiO_3_^25,26^ ferroelectric-based compounds are the better candidates. There had been a various report on the mentioned materials, but achieved EC temperature change (Δ*T*) is very less. It has been simulated for high polarization change, which is the key factor for ECE; associated with K_0.5_Na_0.5_NbO_3_ (KNN) based ceramics^27^ but experimentally there is no report for high positive ECE value so far. It is a ferroelectric material with the perovskite structure which exhibits temperature-dependent spontaneous polarization and possess giant ECE and also the best candidate for solid-state cooling^28,29^. In the present work, we have synthesized lead-free (1-x)K_0.5_Na_0.5_NbO_3_-xLiSbO_3_ (KNN-xLS) nanocrystalline ceramics with 0.015 ≤ x ≤ 0.06 and obtained the highest ECE value and EC responsivity experimentally by an indirect method based on Maxwell’s approach.

## Results

### Phase and microstructure

**Figure 1:**
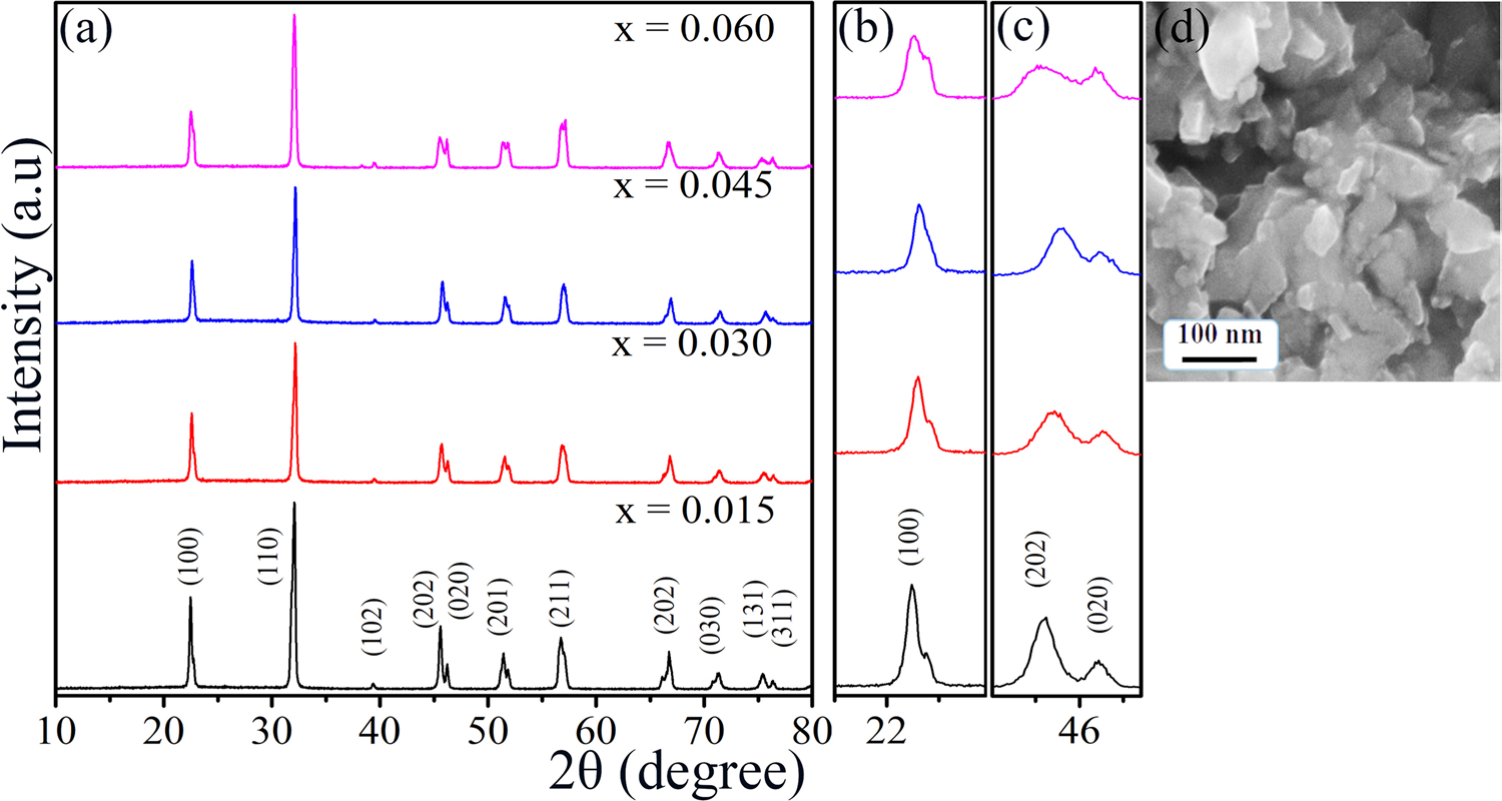
The room temperature XRD patterns of KNN-xLS powder samples (**a**) 2*θ* range 10°–80°, (**b**) enlarge view of (100) peak, (**c**) enlarge view of (202) and (020) peaks, and (**d**) FE-SEM micrograph of KNN-0.03LS ceramic.

The purity and crystallinity of the as-prepared KNN-xLS powder samples were investigated by powder X-ray diffraction (XRD) measurements. Figure 1 shows room temperature XRD patterns from KNN-xLS powder samples, with x = 0.015, 0.03, 0.045 and 0.06, respectively. The crystal structure of KNN is an orthorhombic perovskite structure which is very different from the ilmenite structure of LiSbO_3_^30^. All samples show the perovskite structure and we have not observed any extra or secondary peaks in XRD patterns, indicating that a homogeneous solid solution has formed by diffusing of Li^+^ and Sb^5+^ into the (K_0.5_Na_0.5_)NbO_3_ lattices, where Li^+^ goes to (Na_0.5_K_0.5_)^+^ sites and Sb^5+^ occupies the Nb^5+^ sites. Figure 1b,c show enlarged XRD patterns of (100) and (202/020) peak, respectively. For x ≤ 0.04, the diffraction peaks in Fig. 1 can be indexed to the orthorhombic structure (space group: Bmm2) of KNN with lattice constants of a = 5.69 Å, b = 3.97 Å, c = 5.72 Å and *α* = *β* = *γ* = 90°, which are in good agreement with literature results [i.e. JCPDS-ICDD 2001, Number 71-2171]. The orthorhombic structure is characterized by splitting of (020)/(202) peaks in the 2*θ* range of 44°–47°. The ceramic has an orthorhombic perovskite structure for x ≤ 0.04 which demonstrate the nonprimitive cell, increasing in the concentration of LiSbO_3_ leads to appear the tetragonal phase (primitive) and increases continuously for KNN-xLS. For x = 0.06, the orthorhombic and tetragonal phases coexist also reported by Lin *et al*.^31^. As can be seen in XRD patterns, the diffraction peaks show continuous shifts to higher 2*θ* values with increasing x due to lesser ionic radii of Li^+^ and Sb^5+^ than of the (Na_0.5_K_0.5_)^+^ and Nb^5+^.

The XRD data was used to calculate the average crystallite size using scherrer’s relation, d = 0.9*λ*/*β* cos *θ* ^32^, where d is the average crystallite size, *β* is the full width at half maxima (FWHM) value corrected with instrument broadening and *λ* is the wavelength of Cu-K*α* radiation taken to be 1.54 Å. The calculated crystallite size for KNN-xLS are found to be 35, 41, 53 and 40 nm for x = 0.015, 0.03, 0.045 and 0.06 respectively for (110) peak confirming the nanocrystalline nature of the samples. Figure 1d shows the field emission scanning electron microscope (FE-SEM) micrograph of KNN-0.03LS ceramics. The observed grain size is in the 30–80 nm range, which matches with the obtained size from XRD. The grains are diffused in each other due to surface diffusion and coalescence while high sintering temperature. It also points to the high density of the sample as we have observed in the calculation of density which reflects in the high dielectric constant with high polarization.

### Dielectric study

**Figure 2:**
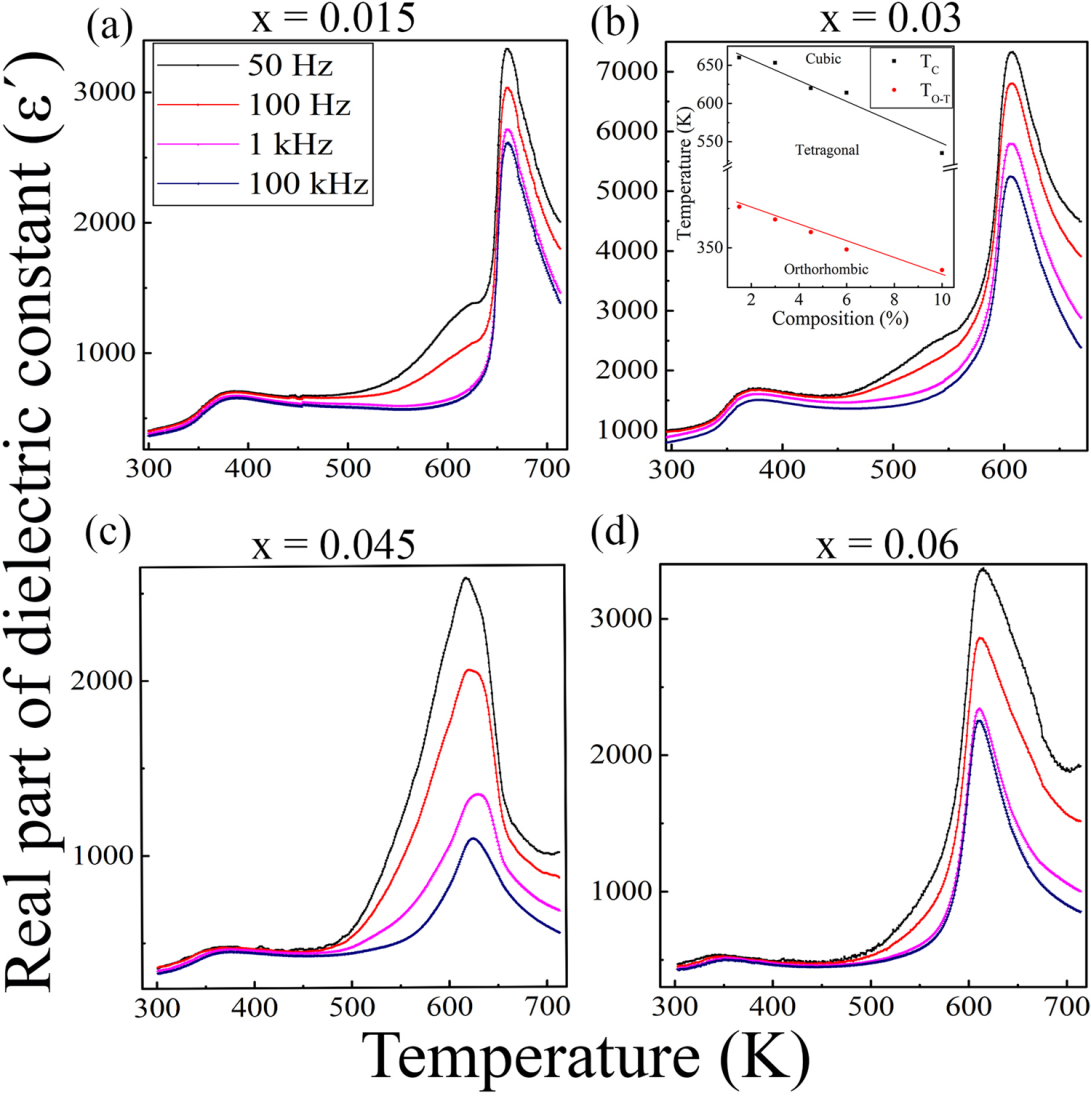
Temperature dependent real part of dielectric constant (*ε*′) of KNN-xLS ceramics for (**a**) x = 0.015, (**b**) x = 0.03, (**c**) x = 0.045, and (**d**) x = 0.06 in the temperature range of 300 K–714 K for frequency 50 Hz to 100 kHz. The inset of (**b**) shows the variation of both transitions T_*C*_ and T_*O*–*T*_ with composition for KNN-xLS ceramics.

The variation of real part of dielectric permittivity (*ε*′) with temperature for different frequencies depicted in Fig. 2a–d for KNN-xLS nanocrystalline ceramics with x = 0.015, 0.03, 0.045 and 0.06, respectively. The *ε*′ was measured for sintered sample in the heating run conditions without any aging process. For pure KNN, the transition from orthorhombic ferroelectric to tetragonal ferroelectric phase (T_*O*–*T*_) occur at 463 K and tetragonal ferroelectric to cubic paraelectric phase (T_*C*_) at 670 K^33^. The addition of LiSbO_3_, shifts T_*C*_ and T_*O*–*T*_ to lower temperature side with retaining the ferroelectric behavior as shown in the inset of Fig. 2b. The T_*C*_ decreases rapidly with increasing LiSbO_3_ than the T_*O*–*T*_. KNN-xLS have ABO_3_ structure, Li^+^ partially substituted to A site ions (K_0.5_Na_0.5_)^+^ and Sb^5+^ to the B site ion Nb^5+^. As it can be noticed the partial substitution of Li^+^ at A-site ions (K_0.5_Na_0.5_)^+^ become a cause of T_*C*_ to increase whereas T_*O*–*T*_ to decrease^34^. Whereas partial submission of Sb^5+^ at B-site ions Nb^5+^ decrease both T_*C*_ and T_*O*–*T*_ fastly^35^, conclusively the temperature falling rates are different for both T_*C*_ and T_*O*–*T*_. The permittivity of KNN-xLS shows the normal ferroelectrics behavior, rather than the relaxor and the character of dependence of dielectric permittivity on frequency resembles the contribution from the increasing electrical conductivity upon increasing of temperature. The observed value of *ε*′ is high for x = 0.03 which is also depicted in the calculation of ECE, a high ECE has been achieved compare to other compositions.

### ECE study

**Figure 3:**
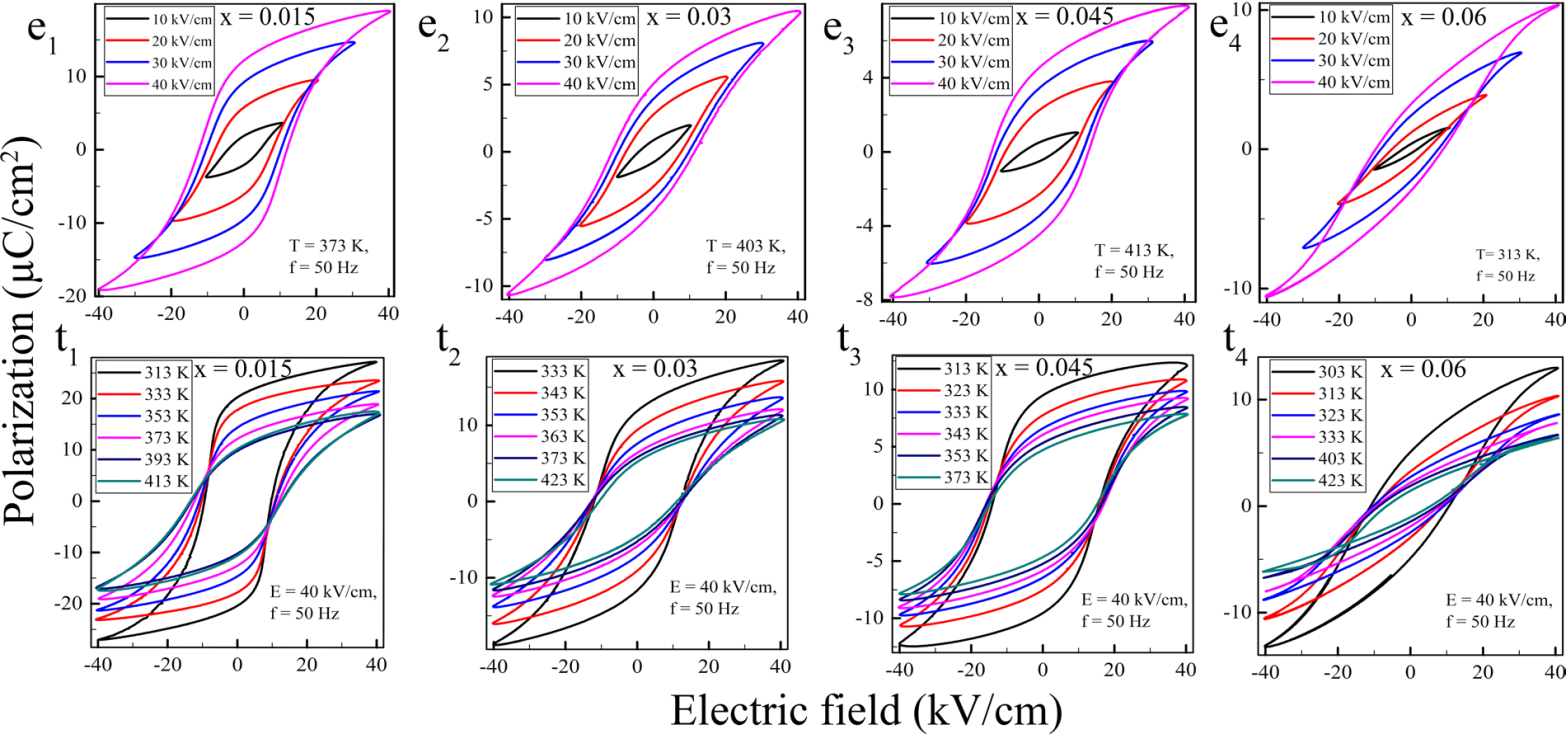
Variation of polarization with electric field at fixed temperature (e_*i*_: i ⊂ $[1,4]$) and with temperature at a fixed field (t_*i*_: i ⊂ $[1,4]$) for KNN-xLS (x = 0.015, 0.03, 0.045, and 0.06) nanocrystalline ceramics.

For calculating the electrocaloric effect of KNN-xLS, the polarization - electric field (P-E) hysteresis loops at 50 Hz were recorded for x = 0.015, 0.03, 0.045 and 0.06 at different temperatures with increment of 10 K and for maximum electric field 40 kV/cm The P-E hysteresis loops have shown in Fig. 3 (e_*i*_: i ⊂ $[1,4]$) for a different field from 10 kV/cm to 40 kV/cm at fixed operating temperature and Fig. 3 (t_*i*_: i ⊂ $[1,4]$) represents the P-E behavior at a fixed field of 40 kV/cm for varying temperatures.

**Figure 4:**
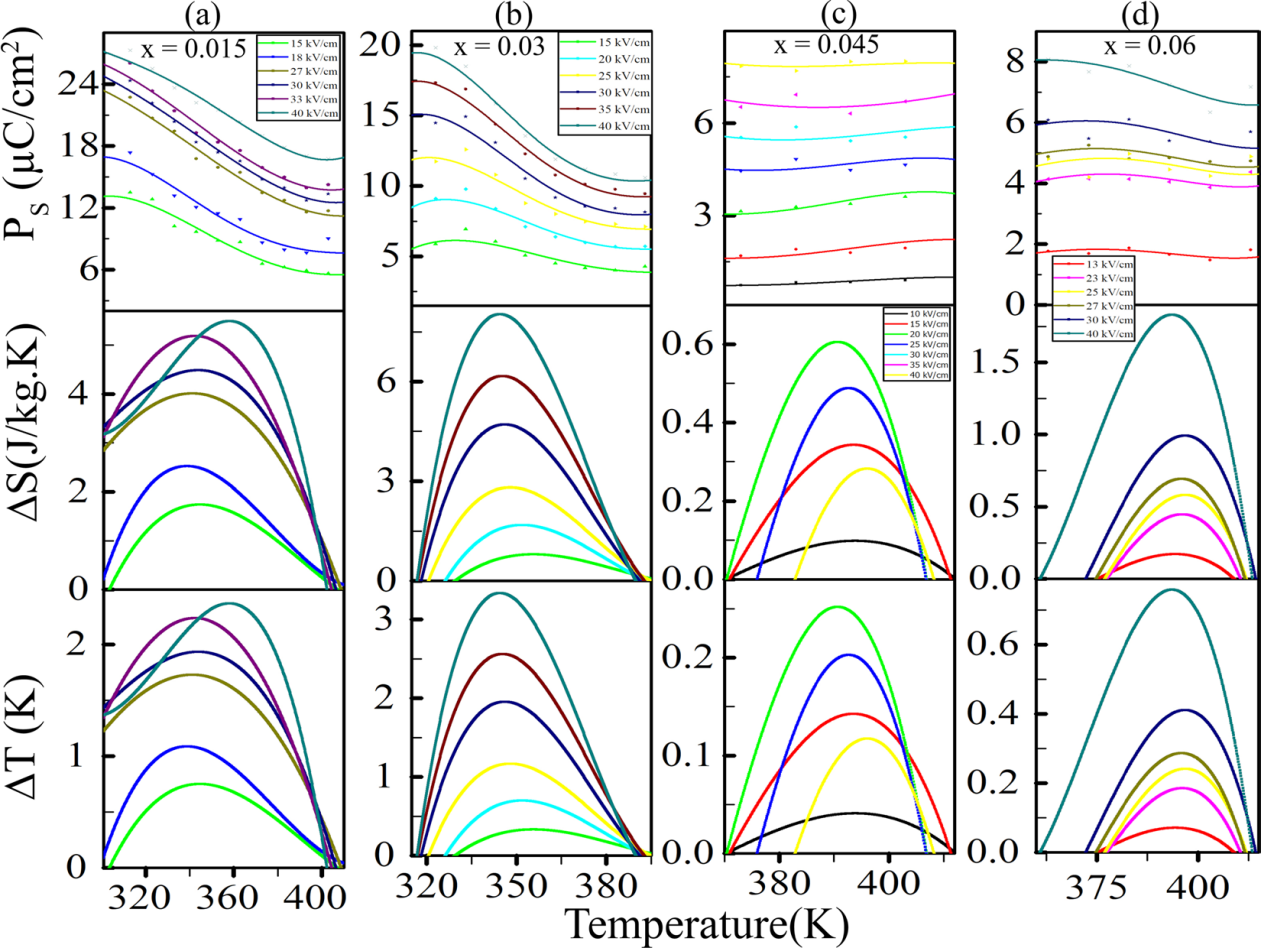
The change in polarization, entropy and electrocaloric temperature as a function of operating temperature for different external electric fields for KNN-xLS nanocrystalline ceramics (**a**) x = 0.015, (**b**) x = 0.03, (**c**) x = 0.045, and (**d**) x = 0.06.

The variation of saturation polarization (P), isothermal entropy change (Δ*S*) and adiabatic temperature change (Δ*T*) with operating temperature for different electric field are shown in Fig. 4a–d for x = 0.015, 0.03, 0.045 and 0.06 respectively. P vs T data is fitted with 4^*th*^ order polynomial, which is also accepted by a number of reports in order to apply Maxwell’s thermodynamic approach to calculate the ECE^1,12^. The key factor to calculate ECE is dP/dT, that can be found by the first order differentiation of P vs T curve. The electrocaloric effect (adiabatic temperature change, Δ*T*) have estimated by indirect approach based on Maxwell’s relation (∂*P*/∂*T*)_*E*_ = (∂*S*/∂*E*)_*T*_, the isothermal entropy (Δ*S*) and the adiabatic temperature change (Δ*T*) at applied electric field E are given by^11,12^.

1 $${\rm{\Delta }}S={\int }_{{E}_{1}}^{{E}_{2}}\,{[\frac{\partial P}{\partial T}]}_{E}dE$$

2 $${\rm{\Delta }}T=-\frac{1}{\rho }\,{\int }_{{E}_{1}}^{{E}_{2}}\,\frac{T}{{C}_{P}}{[\frac{\partial P}{\partial T}]}_{E}dE$$

where E_1_, E_2_ denote the initial and final electric field, *ρ* is the bulk density, C_*P*_ is the specific heat capacity, P is the saturation polarization. E_1_ is taken to be zero and E_2_ is the maximum field. Bulk densities for KNN-xLS; x = 0.015, 0.03, 0.045, 0.06 are 4.22, 4.38, 4.40, 4.39 g/cm^3^ respectively. The specific heat capacity has calculated to be 0.55 J/g.K at 345 K for KNN-0.03LS using differential scanning calorimeter (DSC) measurements and was used for other composition^23,36^. The maximum Δ*S* and Δ*T* are found to be 8.03 J/kg.K and 3.33 K at 345 K for 40 kV/cm using eqs 1 and 2 for KNN-0.03LS. The maximum ECE achieved for 40 kV/cm and higher field was not tried due to the breakdown field which also estimate and limits the dielectric strength. The obtained highest peak corresponding to ECE occurs around the first order phase transition (T_*O*–*T*_) as obtained by the dielectric permittivity measurement shown in Fig. 2b, the peak corresponds to the depolarization state of the sample^37^. Among x = 0.015, 0.03, 0.045, 0.06 composition, the calculated ECE increases from x = 0.015 to x = 0.03 then gets a low value for x = 0.045 and rise again for x = 0.06 shown in the Table 1. Electrocaloric responsivity (Δ*T*/Δ*E*), is the factor to define the variation of the adiabatic temperature change over change in applied field^2^, here E_1_ is taken to be zero and E_2_ is the applied field. The maximum calculated Δ*T*/Δ*E* for KNN-0.03LS is 8.32 × 10^−7^ K.m/V, the trend of variation in Δ*T*/Δ*E* follows the same as for the ECE listed in the Table 1.

**Table 1:** Comparison of EC response for different bulk ceramics.

Composition	Δ*T*(K)	E (kV/cm)	T (K)	Δ*T*/Δ*E* (×10^−7^ K.m/V)	refs
SrBi_2_(Nb_0.2_Ta_0.8_)_2_O_9_	0.19	60	333		^43^
Ba(Zr_0.029_Ti_0.823_)Sn_0.075_O_3_	0.19	8.7	303	2.2	^44^
(Ba_0.95_Ca_0.05_) (Zr_0.1_Ti_0.9_)O_3_	0.205	8	368	2.56	^22^
(Ba_0.8_Ca_0.2_) (Zr_0.08_Ti_0.92_)O_3_	0.22	7.95	377	2.7	^45^
(Bi_0.5_Na_0.5_)_0.94_Ba_0.06_TiO_3_ 4%	0.25	40	375		^17^
1 wt% Li doped (Ba_0.85_Ca_0.15_) (Zr_0.1_Ti_0.9_)O_3_	0.26	20	353	1.64	^16^
(Ba_0.8_Ca_0.2_) (Zr_0.04_Ti_0.96_)O_3_	0.27	7.95	386	3.4	^45^
0.9(K_0.5_Na_0.5_)NbO_3_-0.1Sr(Sc_0.5_Nb_0.5_)O_3_*	0.28	25	357	0.27	^9^
(Ba_0.8_Ca_0.2_)_1−*x*_La_2*x*/3_TiO_3_, x = 0.05	0.30	25	250–400	1.2	^46^
B_0.91_Ca_0.09_Zr_0.14_Ti_0.86_O_3_	0.30	20	333	1.5	^11^
Ba(Zr_0.2_Ti_0.8_)O_3_	0.325	30	310		^21^
0.9(K_0.5_Na_0.5_)NbO_3_-0.1SrTiO_3_*	0.43	40	330	0.7	^42^
[(Bi_1/2_Na_1/2_)_0.94_Ba_0.06_]_1−1.5*x*_La_*x*_TiO_3_, x = 0.03	0.44	42	363		^15^
0.5 mol.% La -doped 0.88Pb(Mg_1/3_Nb_2/3_)O_3_-0.12PbTiO_3_	0.44	40	313		^47^
(Ba_0.835_Ca_0.165_) (Zr_0.09_Ti_0.91_)O_3_	0.46	12	404	3.8	^48^
Ba_0.65_Sr_0.35_TiO_3_	0.49	50	303	1	^25^
0.96(K_0.48_Na_0.52_) (Nb_0.95_Sb_0.05_)O_3_-0.04Bi_0.5_(Na_0.82_K_0.18_)_0.5_ZrO_3_*	0.51	40	350	1.3	^24^
Ba_0.94_Sr_0.06_Ti_0.9_Sn_0.1_O_3_	0.55	20	342	2.75	^49^
BaTi_0.885_Sn_0.105_O_3_	0.61	20	303	3.5	^50^
(Pb_0.88_Sr_0.88_) (Nb_0.08_(Zr_0.53_Ti_0.47_)_0.42_)O_3_	0.65	15	453	4.3	^51^
0.9(0.75Pb(Mg_1/3_Nb_2/3_)O_3_-0.25PbTiO_3_)-0.1PbSnO_3_	0.66	30	373	2.20	^52^
(1-x)Ba(Hf_0.2_Ti_0.8_)O_3_-x(Ba_0.7_Ca_0.3_)TiO_3_, x = 30	0.68	30	340	2.27	^53^
Na_1/2_(Bi_0.98_Gd_0.02_)_1/2_TiO_3_	0.75	90	370	0.8	^18^
BaCe_0.12_Ti_0.88_O_3_	0.8	24	251		^23^
BaTiO_3_	0.9	24	395		^23^
Ba_0.85_Ca_0.15_Ti_0.94_Hf_0.06_O_3_	1.03	35	406		^54^
0.75(Na_0.5_Bi_0.5_)TiO_3_-0.25SrTiO_3_	1.64	50	333	3.3	^19^
(Ba_0.9_Ca_0.1_) (Zr_0.05_Ti_0.95_)O_3_,	1.64	70	403	2.3	^55^
([Bi_1/2_(Na_0.84_K_0.16_)1/2]_0.96_Sr_0.04_) (Ti_0.975_Nb_0.025_)O_3_	1.85	50	305	3.7	^56^
0.85K_0.5_Na_0.5_NbO_3_-0.15SrTiO_3_*	1.9	159	340		^13^
Ba_0.65_Sr_0.35_TiO_3_	2.1	90	303	2.3	^26^
P_0.89_La_0.11_(Zr_0.7_Ti_0.3_)_0.9725_O_3_	2.21	70	423	3.2	^2^
Pb_0.99_Nb_0.02_[(Zr_0.57_Sn_0.43_)_0.92_Ti_0.08_]_0.98_O_3_	2.5	90	361		^57^
0.985(K_0.5_Na_0.5_)NbO_3_-0.015LiSbO_3_*	2.37	40	358	5.9	this work
0.97(K_0.5_Na_0.5_)NbO_3_-0.03LiSbO_3_*	3.33	40	345	8.32	this work
0.955(K_0.5_Na_0.5_)NbO_3_-0.045LiSbO_3_*	0.25	40	390	0.63	this work
0.94(K_0.5_Na_0.5_)NbO_3_-0.06LiSbO_3_*	0.76	40	393	1.9	this work

(*KNN-based ceramics).

**Figure 5:**
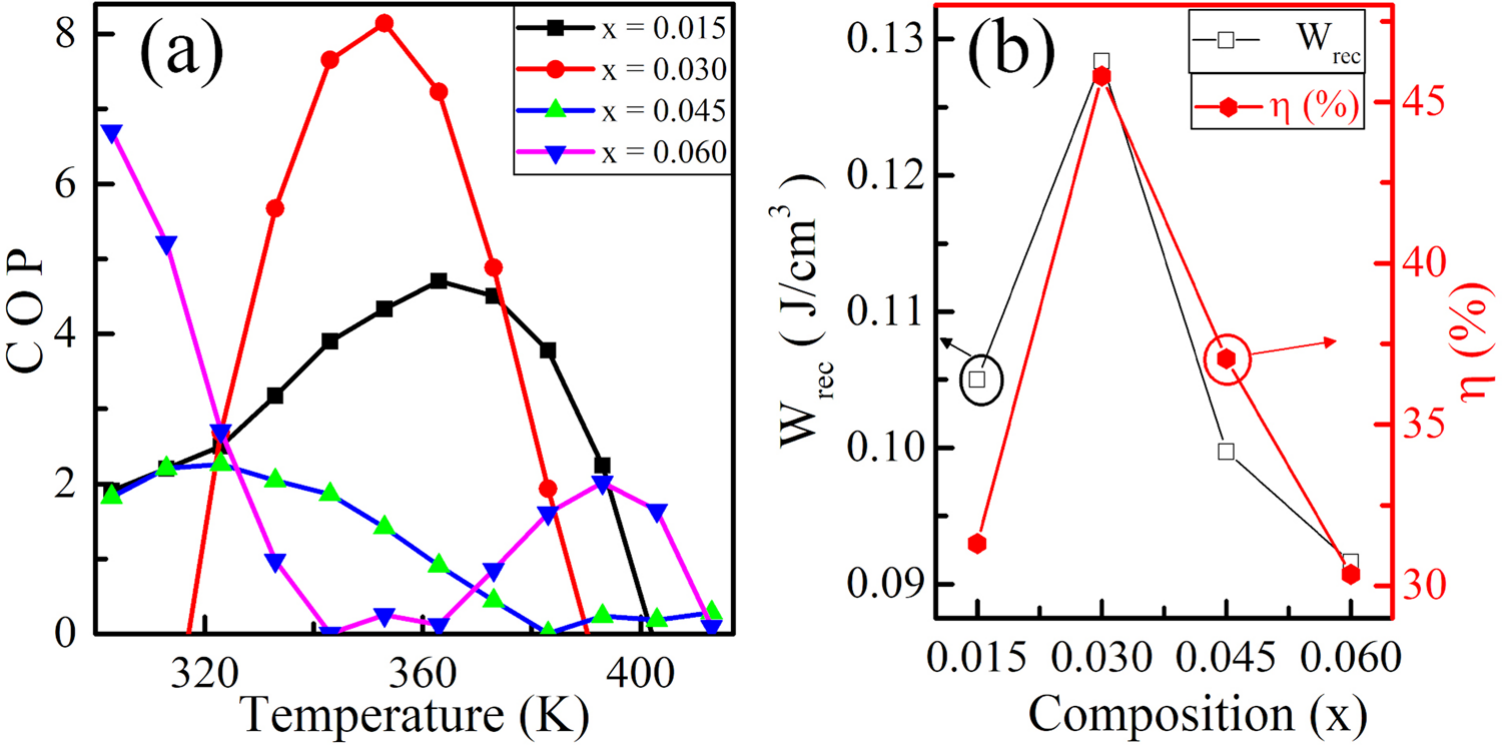
Variation of (**a**) COP with temperature (**b**) W_*rec*_ and *η* (%) with composition for KNN-xLS (x = 0.015, 0.03, 0.045, and 0.06) nanocrystalline ceramics.

For ferroelectric ceramics, the electrical energy storage density and the efficiency can be obtained from P-E loops by the eqs 3, 4 and 5 ^38^.

3 $${W}_{rec}={\int }_{{P}_{r}}^{{P}_{max}}\,EdP$$

4 $${W}_{total}={\int }_{0}^{{P}_{max}}\,EdP$$

5 $$\eta =\frac{{W}_{rec}}{{W}_{total}}\times \mathrm{100 \% }$$

where E is the applied field, P is the polarization, W_*rec*_ is the recoverable energy, W_*total*_ is total energy and *η* is the efficiency. Figure 5 shows the variation of W_*rec*_, *η* for all the value of x in KNN-xLS. The calculated maximum W_*rec*_ is 0.128 J/cm^3^ for KNN-0.03LS and efficiency (*η*) is 46% which is higher than various other lead - free ceramics. The coefficient of performance (COP), a factor to evaluate the cooling performance can be calculated as COP = |Q|/W_*total*_, Where Q is *T*.Δ*S*; a ratio of extracted heat and the input work. The maximum COP, 8.14 at 353 K obtained for KNN-0.03LS shown in Fig. 5a and it is larger than for the other compositions.

## Discussion

The recorded P-E loops at each temperature and the field are not slim, which is also an evident for non-relaxor behavior that is in connection with the variation of dielectric permittivity with temperature and it also represent the coarse grain microstructure for the material^39^. The size of the grain impacts the breakdown strength (BDS) over the applied field, which is described by E $\propto$ G^−*a*^, where E is the breakdown field, G is the grain size and ‘a’ is a constant^40^. It states that smaller grain increases the BDS which further results in high P and high ECE along with good energy storage capacity^41^. The net achieved polarization is induced by poling and the symmetry occurs in loops because of lacking of pining defect dipoles, increasing in space charge polarization is the reason for the change in shape of the P-E loops for compositions shown in Fig. 3(e_1_ to e_4_). At the lower field, the P-E loop is not in the saturated state and rising the field makes the P-E loop saturated, which resembles the higher energy density state and the increasing dielectric strength of the material. The saturated P-E hysteresis loop is due to the coexistence of both orthorhombic and tetragonal phases near the transition temperature. As rising in the temperature, the polarization decreases for all the compositions which indicates vanishing the dipole arrangement due to thermal agitation.

For the ECE measurement, the Maxwell relation has been used which is widely accepted with the 4^*th*^ order polynomial fitted P vs T curve^2^. The calculated maximum ECE found for the case of KNN-0.03LS with the reason for having high real part of the dielectric constant which implies the high mobility of domains. Whereas for the x = 0.015, 0.045, 0.06 it is less than the 0.03 which is also depicted in the plot for real part of the dielectric permittivity shown in Fig. 2. This behavior of ECE is in relation with the obtained dielectric constant corresponding to their compositions concludes that ceramics with high dielectric constant exhibits Δ*T* more. As listed in the Table 1, KNN-0.03LS composition has large ECE value among all lead-free nanocrystalline ceramics reported so far in any mode of measurements.

Even if for the case of thin/thick films, the ECE obtained to be more, but the electrocaloric responsivity (Δ*T*/Δ*E*) is less than for the bulk due to thin film needs high field for ECE. Thin films have high breakdown field strength *i*.*e*. higher the sustainability whereas bulk ceramics have less breakdown strength due to some extrinsic factors (voids, interfaces, defects, etc). KNN-0.03LS also shows a high coefficient of performances (COP) among other lead-free ceramics, it shows the high refrigeration capacity for the material. The recoverable energy storage capacity and efficiency is higher among many other lead-free ceramics.

In summary, The electrocaloric effect of (1 − x)K_0.5_Na_0.5_NbO_3_-xLiSbO_3_ nanocrystalline ceramics with x = 0.015, 0.03, 0.045 and 0.06 are evaluated using Maxwell’s thermodynamic relation. The microstructures and dielectric permittivity of the sample were studied and discussed in relation with ECE. The remarkable electrocaloric efficiency (Δ*T*/Δ*E*), COP have been observed with the effective change of the temperature. The calculated maximum ECE peak has found near phase transition temperatures. It was found to be with high energy storage capacity and COP. Conclusively KNN-0.03LS become the more potential candidate for refrigeration as micro cooler and in energy storage applications, etc.

## Methods

### Preparation of samples

A conventional solid-state route had been followed in the synthesis of (1-x)K_0.5_Na_0.5_NbO_3_-xLiSbO_3_ samples^9,42^. The high purity ($\gg$99%) starting precursors K_2_CO_3_ (Hi-Media), Na_2_CO_3_ (Hi-Media), Nb_2_O_5_ (Hi-Media), C_2_H_3_LiO_2_.2H_2_O (Sigma) and C_6_H_9_O_6_Sb (Hi-Media) were used. The stoichiometric, homogenized mixture of precursors were grinded using mortar and pestle (agate) for 8 h till to achieving the uniform small size of the mixture. The dried mixed precursor powder was calcined at 1153 K for 5 h. The calcined powder was uniaxially pressed (~125 kN) into the disk form of 10 mm diameter with 1 mm thickness using steel die after mixing with 5 wt% polyvinyl alcohol solution prepared in water and compacts were sintered further for 1343–1383 K for 4 h in ample oxygen using the double crucible method to suppress the volatile nature of alkali oxides.

### Characterization

X-ray diffraction (XRD) data of sintered powder was obtained using a X-ray diffractometer (Rigaku mini Flex 600, Japan: *λ* = 1.54 Å) with Cu-K*α* radiation. The surface morphology has been observed by field-emission scanning electron microscopy (FE-SEM, LYRA3-TESCAN). The density of the samples were measured using the Archimedes method with di-ionized water. The dielectric permittivity measurements have performed for the frequency 50 Hz–1 MHz in the temperature range of 303 K–773 K by an impedance analyzer (E4990A, Keysight Technologies) at 0.50 V osc voltage and zero bias voltage. Furthermore, the Polarization-electric field (P-E) characterization was carried out using a P-E Loop Tracer (Marine India). For the electric measurements, both the surfaces of the pallet was layered by a uniform thin silver paste and dried at 773 K for 25 minutes. The specific heat capacity (C_*p*_) was calculated using a differential scanning calorimeter (Mettler Toledo DSC-3).
